# Machine Learning Guidance for Connection Tableaux

**DOI:** 10.1007/s10817-020-09576-7

**Published:** 2020-09-05

**Authors:** Michael Färber, Cezary Kaliszyk, Josef Urban

**Affiliations:** 1 University of Innsbruck, Innsbruck, Austria; 2 Czech Technical University in Prague, Prague, Czech Republic

**Keywords:** Connection tableaux, Internal guidance, Monte Carlo

## Abstract

Connection calculi allow for very compact implementations of goal-directed proof search. We give an overview of our work related to connection tableaux calculi: first, we show optimised functional implementations of connection tableaux proof search, including a consistent Skolemisation procedure for machine learning. Then, we show two guidance methods based on machine learning, namely reordering of proof steps with Naive Bayesian probabilities, and expansion of a proof search tree with Monte Carlo Tree Search.

## Introduction

Connection calculi enable goal-directed proof search in a variety of logics. Connections were considered among others for classical first-order logic [49], for higher-order logic [3] and for linear logic [23].

An important family of connection provers for first-order logic is derived from leanCoP [57, 62]. leanCoP was inspired by leanTAP [5], which is a prover based on free-variable semantic tableaux. leanTAP popularised *lean theorem proving*, which uses Prolog to maximise efficiency while minimising code. The compact Prolog implementation of *lean theorem provers* made them attractive for experiments both with the calculus and with the implementation. For example, leanCoP has been adapted for intuitionistic (ileanCoP [56]), modal (MleanCoP [60]), and nonclausal first-order logic (nanoCoP [61]). The intuitionistic version of leanCoP [56] became the state-of-art prover for first-order problems in intuitionistic logic [67]. A variant of leanCoP with interpreted linear arithmetic (leanCoP-$\varOmega$) won the TFA division of CASC-J5 [77]. Various implementation modifications can be performed very elegantly, such as search strategies, scheduling, restricted backtracing [58], randomization of the order of proof search steps [66], and internal guidance [38, 83].

We have used connection provers from the leanCoP family as a basis for experiments with *machine learning* (see Sect. 5) and *proof certification* [41]. For these applications, we implemented connection provers in functional instead of logic programming languages. There are several reasons: First, a large number of interactive theorem provers (ITPs), such as HOL Light [28], HOL4 [74], Isabelle [86], Coq [8], and Agda [16] are written in functional programming languages, lending themselves well to integration of functional proof search tactics. Second, several machine learning algorithms such as Naive Bayes and k-NN have been implemented efficiently for ITPs in functional languages [15, 39]. Third, we achieve better performance with functional-style implementations, which is important to compensate for the performance penalty incurred by machine learning.

In this paper we develop an integration of internal guidance based on machine learning and Monte Carlo methods in connection-style proof search. The contributions described in this paper are:

- We implement proof search based on clausal and nonclausal connection tableaux calculi in functional programming languages, improving performance upon previous Prolog-based implementations, see Sect. 3.
- We show a method to order proof search steps by using a Naive Bayes classifier based on previous proofs, see Sect. 4.
- We use Monte Carlo Tree Search to guide connection proof search, see Sect. 5. To this end, we propose and evaluate several proof state evaluation heuristics, including two that learn from previous proofs.

The paper combines, compares, and extends our works presented at LPAR 2015 [38] and CADE 2017 [22]. The techniques added over the conference versions include: consistent Skolemisation applicable also for nonclausal proof search and efficient functional-style implementation of proof search in clausal and nonclausal connection calculi.^1^

## Connection Calculi

Connection calculi provide a goal-oriented way to search for proofs in classical and nonclassical logics [57]. Common to these calculi is the concept of connections $\left\{ P, \lnot P\right\}$ between literals *P* and $\lnot P$, which correspond to closing a branch in the tableaux calculus [26]. Among these calculi are the connection method [9, 10], the connection tableau calculus [48], and model elimination [51].

In this section, we introduce the *clausal connection calculus* that we will use throughout the paper. As this calculus has a small set of rules, it lends itself very well to machine learning. For a description of the rules of the *nonclausal connection calculus*, we refer to [59].

The connection calculi in this paper operate on *matrices*, where a matrix is a set of clauses. In the clausal connection calculus, a clause is a set of literals. In the nonclausal calculus, clauses do not only contain literals, but also matrices, giving rise to a nested structure. We use the symbols *M* for a matrix, *C* for a clause, *L* for a literal, *x* for a variable, and $\mathbf{x}$ for a sequence of variables, as in $\forall \mathbf{x}. P(\mathbf{x})$. A substitution $\sigma$ is a mapping from variables to terms. The *complement* $\overline{L}$ is *A* if *L* has the shape $\lnot A$, otherwise, $\overline{L}$ is $\lnot A$. A $\sigma$*-complementary connection* $\left\{ L, L'\right\}$ exists if $\sigma \overline{L} = \sigma L'$. Given a relation *R*, its transitive closure is denoted by $R^+$ and its transitive reflexive closure by $R^*$.

We now give a definition of the common parts of the clausal and nonclausal connection calculi.

### Definition 1

(*Connection Calculus, Connection Proof*) The words of a *connection calculus* are tuples $\langle C, M, Path \rangle$, where *C* is a clause, *M* is a matrix, and *Path* is a set of literals called the *active path*. *C* and *Path* can be empty, denoted $\varepsilon$. In the calculus rules, $\sigma$ is a term substitution and $\left\{ L, L'\right\}$ is a $\sigma$-complementary connection. The substitution $\sigma$ is global (or *rigid*), i.e. it is applied to the whole derivation. A *connection proof* for $\langle C, M, Path \rangle$ is a derivation in a connection calculus for $\langle C, M, Path \rangle$ in which all leaves are axioms. A connection proof for *M* is a connection proof for $\langle \varepsilon , M, \varepsilon \rangle$.

**Fig. 1:**
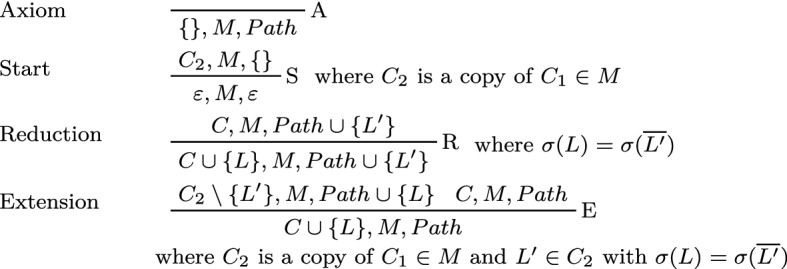
Clausal connection calculus rules

To complete the definition of the clausal connection calculus, we present the calculus rules in Fig. 1.

Given an order <, we can write sets as ordered sequences $[X_1, \dots , X_n]$, where for all $i < n$, $X_i < X_{i+1}$. Clauses and matrices can thus be shown as horizontal and vertical sequences, respectively.

### Example 1

Consider the following formula *F* and its prenex conjunctive normal form $F'$. We will show that $F'$ implies $\bot$:

$$\begin{aligned} F&= Q \wedge P(a) \wedge \forall x. (\lnot P(x) \vee (\lnot P(s^2 x) \wedge (P(sx) \vee \lnot Q))) \\ F'&= \forall x. (Q \wedge P(a) \wedge (\lnot P(x) \vee \lnot P(s^2 x)) \wedge (\lnot P(x) \vee P(sx) \vee \lnot Q)) \end{aligned}$$

For brevity, we write *sx* for *s*(*x*) and $s^2 x$ for *s*(*s*(*x*)). The clausal matrix $M'$ corresponds to $F'$:

$$\begin{aligned} M' = \left[ [Q]\; [P(a)] \left[ \begin{matrix} \lnot P(x) \\ \lnot P(s^2 x) \end{matrix}\right] \left[ \begin{matrix} \lnot P(x) \\ P(sx) \\ \lnot Q \end{matrix}\right] \right] \end{aligned}$$

**Fig. 2:**
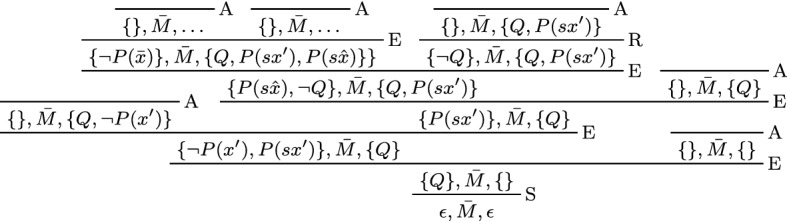
Clausal connection proof

A formal proof for $M'$ in the clausal connection calculus is given in Fig. 2.

Soundness and completeness have been proved both for the clausal [48] and for the nonclausal connection calculus [59]. We will discuss practical functional-style implementations of proof search for both calculi in Sect. 3.

## Functional-Style Connection Prover

In this section, we develop an efficient implementation of a connection prover for classical first-order logic in a functional programming language. The resulting implementation will be the basis for all experiments in the remainder of the paper.

The connection prover performs the following tasks. Given a classical first-order logic problem, it creates a matrix for the problem, see Sect. 3.1. The matrix is then used to build an index that provides an efficient way to find connections during proof search, see Sect. 3.3. Finally, proof search with iterative deepening is performed, see Sect. 3.4.

### Problem Preprocessing

In this section, we show how the prover transforms problems into formulas and processes them to yield a matrix. We focus on first-order logic problems represented as a set of axioms $\{A_1, \dots , A_n\}$ together with a conjecture *C*, where all axioms and the conjecture are closed formulas. The goal is to show that the axioms imply the conjecture. For convenience, in the actual implementation we use the TPTP format [76] as input. Each parsed input problem is transformed according to the following procedure. Only the steps 2 and 6 differ in comparison with the original Prolog implementations of leanCoP and nanoCoP [57, 61].

1. The conjecture *C* is combined with the axioms $\{A_1, \dots , A_n\}$ to form the new problem $(A_1 \wedge \dots \wedge A_n) \rightarrow C$ (just *C* if no axioms are present).
2. Constants and variables are mapped to integers, to enable more efficient lookup and comparison during the proof search, as needed e.g. for fast unification.
3. As the connection tableaux calculi considered in this paper do not have special rules for equality, equality axioms are added to the problem if equality appears in the original problem. The axioms are symmetry, reflexivity, and transitivity 
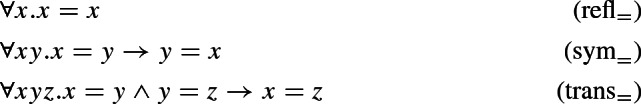
 as well as congruence:
  For every *n*-ary function *f*, the formula $x_1 = y_1 \rightarrow \dots \rightarrow x_n = y_n \rightarrow f(x_1, \dots , x_n) = f(y_1, \dots , y_n)$ is introduced.
  For every *n*-ary predicate *P*, the formula $x_1 = y_1 \rightarrow \dots \rightarrow x_n = y_n \rightarrow P(x_1, \dots , x_n) \rightarrow P(y_1, \dots , y_n)$ is introduced.
4. If the formula has the shape $P \rightarrow C$, then it is transformed to the equivalent $(P \wedge \#) \rightarrow (C \wedge \#)$. $\#$ is a marker that can be understood to be equivalent to $\top$. It allows proof search to recognise clauses stemming from the conjecture [57, section 2.1].
5. Implications and equivalences are expanded, e.g. $A \rightarrow B$ becomes $\lnot A \vee B$.
6. Quantifiers are pushed inside so that their scope becomes minimal.
7. The formula is negated (to perform a proof by refutation) and converted to negation normal form.
8. The formula is reordered so that smaller clauses are processed earlier. In nanoCoP, the size of a formula is
  $$\begin{aligned} {{\,\mathrm{\text {paths}}\,}}(t) ={\left\{ \begin{array}{ll} {{\,\mathrm{\text {paths}}\,}}(t_1) \times {{\,\mathrm{\text {paths}}\,}}(t_2) &{}\quad \text {if }\quad t = t_1 \wedge t_2 \\ {{\,\mathrm{\text {paths}}\,}}(t_1) + {{\,\mathrm{\text {paths}}\,}}(t_2) &{}\quad \text {if }\quad t = t_1 \vee t_2 \\ {{\,\mathrm{\text {paths}}\,}}(t_1) &{} \quad \text {if } t = \forall x. t_1 \text { or }\quad t = \exists x. t_1 \\ 1 &{}\quad \text {if }\quad t \text { is a literal} \end{array}\right. } \end{aligned}$$
  and for any subformula $t_1 \wedge t_2$ or $t_1 \vee t_2$, if ${{\,\mathrm{\text {paths}}\,}}(t_1) > {{\,\mathrm{\text {paths}}\,}}(t_2)$, then $t_1$ and $t_2$ are exchanged.
9. The formula is Skolemised. For machine learning, we use consistent Skolemisation as discussed in Sect. 3.2 instead of outer Skolemisation as performed in the original Prolog version.

#### Example 2

Consider the axioms 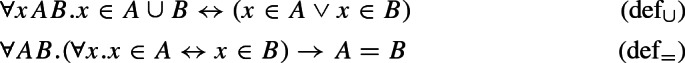 that we want to use to prove 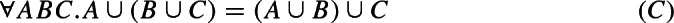 The problem is preprocessed as follows:

1. The axioms $A \equiv \text {def}_{\cup } \wedge \text {def}_{=}$ and the conjecture are combined, resulting in $A \rightarrow C$.
2. Constants and variables are mapped to integers, e.g. $\{$ “$\in$” $\mapsto 0$, “$\cup$” $\mapsto 1$, “$=$” $\mapsto 2\}$ and $\{$ “*x*” $\mapsto 0$, “*A*” $\mapsto 1$, “*B*” $\mapsto 2 \}$. We will continue the presentation of this example with the original representation.
3. Congruence axioms are generated for all constants, i.e. “$\in$” and “$\cup$”: 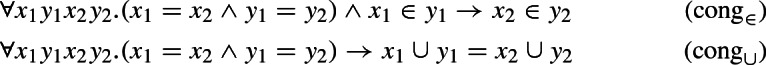 The combination of all equality axioms is 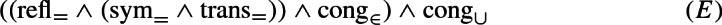 and the resulting formula is $E \wedge A \rightarrow C$.
4. The conjecture is marked, resulting in $((E \wedge A) \wedge \#) \rightarrow (\# \wedge C)$.
5. Implications and equivalences are unfolded. Among others, this transforms 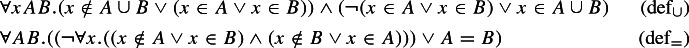 The resulting formula is $\lnot ((E \wedge A) \wedge \#) \vee (\# \wedge C)$.
6. Pushing quantifiers inside transforms for example 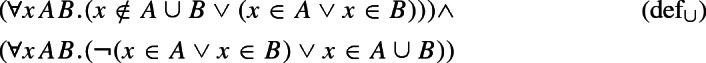
7. The whole formula is negated and converted to negation normal form. In particular, the negation of the conjecture is 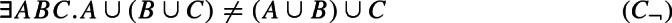 and the resulting formula is $((E \wedge A) \wedge \#) \wedge (\lnot \# \vee C_\lnot )$.
8. Reordering of the formula yields among others 
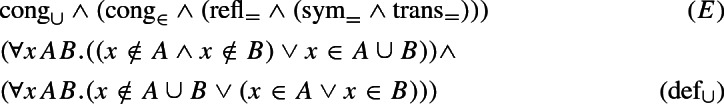
 and the resulting formula is $(\lnot \# \vee C_\lnot ) \wedge (\# \wedge ((\text {def}_= \wedge \text {def}_\cup ) \wedge E))$. Note that the equality axioms move to the end of the formula, so they are being processed last.
9. Skolemisation replaces existentially quantified variables by Skolem terms and removes existential quantifiers. For example, the Skolemised negated conjecture is 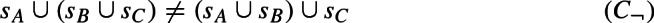 where $s_A$, $s_B$, and $s_C$ are nullary Skolem functions. We explain Skolemisation in more detail in Sect. 3.2.

The matrix is built from the resulting formula. For the clausal connection prover, this involves a transformation of the formula into clausal normal form. The *standard transformation* applies distributivity rules of the shape $A \wedge (B \vee C) \equiv (A \vee B) \wedge (A \vee C)$ to the formula until it is in conjunctive normal form. In the worst case, this transformation makes the formula grow exponentially. To avoid this, the *definitional transformation* introduces new symbols [58, 63, 80]. Similarly to Skolemisation, the introduced symbols should be consistent across different problems, which is achieved by using a normalised string representation of the clause literals as new symbol names. For the nonclausal connection prover, no clausification is required, as the formula can be directly transformed into the nonclausal matrix. For both clausal and nonclausal matrices, the polarity of literals is encoded by the sign of the integer representing the predicate symbol.

### Consistent Skolemisation

The Skolemisation of a formula $\varDelta$ replaces existentially quantified variables occurring in $\varDelta$ by newly introduced function symbols called Skolem functions, yielding a formula equisatisfiable to $\varDelta$ without existential quantifiers. Skolemisation may introduce distinct Skolem functions when a single Skolem function would have sufficed. For example, a subformula $\exists x. P(x)$ of two formulas $\varDelta _1$ and $\varDelta _2$ may be Skolemised to $P(s_1)$ in $\varDelta _1$ and to $P(s_2)$ in $\varDelta _2$, such that $s_1 \ne s_2$. This makes it difficult to spot in hindsight that $s_1$ and $s_2$ were produced from equivalent subformulas. For machine learning, however, when we learn something about a formula containing a Skolem function, such as $P(s_1)$, we wish to transfer this knowledge to a different formula where the same formula was Skolemised to $P(s_2)$. To solve this problem, we present a new Skolemisation method that introduces Skolem functions “consistently”. In general, a consistent Skolemisation method can instantiate different existentially quantified variables with the same Skolem functions under certain conditions. For example, a consistent Skolemisation method could ensure for the example above that $s_1 = s_2$.

Consistent Skolemisation methods have been studied in the context of the $\delta$-rule in tableaux methods [6]. The Skolem terms introduced by such methods may lead to rather large formulas unless techniques such as structure sharing are used [24]. However, in our setting, such techniques would complicate the parallel execution of several prover instances and require the adaption of both the prover and the machine learning methods. We propose a new consistent Skolemisation method which produces reasonably small Skolem functions without relying on structure sharing.

We assume that the formulas in this section are in negation normal form. A position *p* is a sequence $p_1 \dots p_n$, where every $p_i$ is either 0 or 1. The empty sequence $\varepsilon$ denotes the root position, and *pq* is the concatenation of two positions *p* and *q*. The subformula of *F* at the position *p*, denoted as $F|_p$, is defined as follows: $F|_\varepsilon = F$, and if $F = \exists x. G$ or $F = \forall x. G$, then $F|_{0p} = G|_p$, and if $F = G_1 \wedge G_2$ or $F = G_1 \vee G_2$, then $F|_{0p} = G_1|_p$ and $F|_{1p} = G_2|_p$.

The sequence of free variables of *F* is denoted by ${{\,\mathrm{\mathcal {FV}ar}\,}}(F)$. The order of the sequence ${{\,\mathrm{\mathcal {FV}ar}\,}}(F)$ must not depend on the names of the variables; i.e. for any bijective substitution $\sigma$, if $\sigma F = G$, then $\sigma {{\,\mathrm{\mathcal {FV}ar}\,}}(F) = {{\,\mathrm{\mathcal {FV}ar}\,}}(G)$. The sequence of existentially/universally quantified variables of a formula *F* along *p* is ${{\,\mathrm{\mathcal {V}ar}\,}}_Q(F, p) = \bigcup _{i < |p|} \left\{ x \mid \varDelta |_{p_1 \dots p_i} = Q x. G\right\}$, where the sequence is ordered by ascending *i* and $Q \in \left\{ \forall , \exists \right\}$. For example, if $\varDelta = \forall x. \exists y z. P(x, y, z)$ and $p = 00$, then $\varDelta |_p = \exists z. P(x, y, z)$, ${{\,\mathrm{\mathcal {V}ar}\,}}_{\forall }(\varDelta , p) = \left[ x\right]$, and ${{\,\mathrm{\mathcal {V}ar}\,}}_{\exists }(\varDelta , p) = \left[ y\right]$.

To describe multiple Skolemisation methods, we introduce a Skolemisation operator $S_f(\sigma , \varDelta , p)$. This operator is parametrised by a Skolemisation function $f(x, \sigma , \varDelta , p) =\sigma '$, which yields for a formula $\sigma (\varDelta |_p) =\exists x. F$ an equisatisfiable formula $\sigma ' (\varDelta |_{p0})$.^2^ The operator $S_f(\sigma , \varDelta , p)$ returns the *f*-Skolemisation of $\sigma (\varDelta |_p)$. It follows that the *f*-Skolemisation of $\varDelta$ is $S_f(\emptyset , \varDelta , \varepsilon )$. The purpose of a Skolemisation function is to eliminate a single existential quantifier, whereas the Skolemisation operator uses the Skolemisation function to eliminate all existential quantifiers of a formula.

$$\begin{aligned} S_f(\sigma , \varDelta , p) ={\left\{ \begin{array}{ll} S_f(f(x, \sigma , \varDelta , p), \varDelta , p0) &{}\quad \text {if }\quad \varDelta |_p = \exists x. F \\ \forall x. S_f(\sigma , \varDelta , p0) &{}\quad \text {if }\quad \varDelta |_p = \forall x. F \\ S_f(\sigma , \varDelta , p0) \wedge S_f(\sigma , \varDelta , p1) &{} \quad \text {if }\quad \varDelta |_p = F_1 \wedge F_2 \\ S_f(\sigma , \varDelta , p0) \vee S_f(\sigma , \varDelta , p1) &{}\quad \text {if }\quad \varDelta |_p = F_1 \vee F_2 \\ \sigma \varDelta |_p &{} \quad \text {if }\quad \varDelta |_p = A \text { or } \varDelta |_p = \lnot A \end{array}\right. } \end{aligned}$$

The Skolemisation function for inner Skolemisation is $IS(x, \sigma , \varDelta , p) = \sigma \cup \left\{ x \mapsto s(\mathbf{y})\right\}$ with *s* denoting a fresh Skolem function symbol and $\mathbf{y} ={{\,\mathrm{\mathcal {FV}ar}\,}}(\sigma (\varDelta |_p))$. This performs inner Skolemisation from left to right, to avoid nesting of Skolem functions [54]. While producing small formulas, this is not a consistent Skolemisation method, as all different existentially quantified variables are mapped to different Skolem functions.

An alternative Skolemisation method uses epsilon notation [29]. The critical axiom of the epsilon calculus is $P(t) \rightarrow P(\epsilon x. P(x))$ from which one can derive $\exists x. P(x) \leftrightarrow P(\epsilon x. P(x))$. The Skolemisation function for epsilon-Skolemisation is then $\epsilon S(x, \sigma , \varDelta , p) = \sigma \cup \left\{ x \mapsto \epsilon x. \sigma (\varDelta |_{p0})\right\}$. Epsilon-Skolemisation maps the subformulas $\exists x. P(x)$ from the introductory example to the same term, namely $P(\epsilon x. P(x))$, therefore it is a consistent Skolemisation method. However, epsilon-Skolemisation requires an extension of first-order logic. Furthermore, like previous consistent Skolemisation methods, it can yield exponentially large formulas unless structural sharing is used.

The key to obtaining a consistent Skolemisation method for our setting is to combine inner Skolemisation with epsilon-Skolemisation. We can express any Skolem term $s(\mathbf{y})$ introduced by inner Skolemisation by some epsilon term $\epsilon x. F$ introduced by epsilon-Skolemisation. We will show a consistent Skolemisation method that uses this correspondence, by introducing the same Skolem terms whenever their underlying epsilon terms are alpha-equivalent.

We now show how to consistently Skolemise a subformula $\exists x. F$ of $\varDelta$ at position *p*. We will refer to variables that are existentially quantified in $\varDelta$ as existential variables and to variables that are universally quantified in $\varDelta$ as universal variables. Our consistent Skolemisation proceeds in three steps: First, we obtain the smallest subformula $F_{\min }$ of $\varDelta$ that contains $\varDelta |_p$ and contains no free existential variables. The minimality of $F_{\min }$ serves to maximise the number of equivalent existential variables being mapped to the same Skolem function, which is important for learning. Next, we obtain the position *q* of $\varDelta |_p$ in $F_{\min }$. The idea is that encoding the combination of $F_{\min }$ and *q* in the name of the Skolem function suffices to characterise $\varDelta |_p$, allowing to reconstruct the epsilon term $\epsilon x. F$. Finally, we obtain the arguments of the Skolem function.

1. We show how to obtain $F_{\min }$. For this, we determine the path $p_1 \dots p_m$ that is the longest prefix of *p* such that $\varDelta |_{p_1 \dots p_m}$ does not contain free existential variables. We obtain this by $m = \max \left\{ i \mid {{\,\mathrm{\mathcal {V}ar}\,}}_{\exists }(\varDelta , p_1 \dots p_i) \cap {{\,\mathrm{\mathcal {FV}ar}\,}}(\varDelta |_{p_1 \dots p_i}) = \emptyset \right\}$, from which we can obtain $F_{\min } = \varDelta |_{p_1 \dots p_m}$. Because $F_{\min }$ may contain free universal variables $\mathbf{v}_m = {{\,\mathrm{\mathcal {FV}ar}\,}}(F_{\min })$, we abstract over $\mathbf{v}_m$ to obtain a closed term $F_\lambda =\lambda \mathbf{v}_m. F_{\min }$ that can be alpha-normalised. We call $F_\alpha$ the alpha-normalisation of $F_\lambda$.
2. We obtain the position *q* of $\varDelta |_p$ in $F_{\min }$ by the equation $p = p_1 \dots p_m q$. We also obtain the universal variables free in $\varDelta |_p$ by $\mathbf{v}_q = {{\,\mathrm{\mathcal {V}ar}\,}}_{\forall }(F_{\min }, q) \cap {{\,\mathrm{\mathcal {FV}ar}\,}}(F_{\min }|_q)$.
3. We determine the arguments of the Skolem term to be $\mathbf{y} = \mathbf{v}_m \mathbf{v}_q$.

Putting everything together, the Skolemisation function for our consistent Skolemisation is $CS(x, \sigma , \varDelta , p) = \sigma \cup \left\{ x \mapsto s^{F_\alpha }_{q}(\mathbf{y})\right\}$, where $s^F_p$ denotes a first-order function symbol that carries in its name the formula *F* as well as the position *p*.

#### Example 3

Our consistent Skolemisation of the introductory example $\exists x. P(x)$ is $P(s^{\exists x. P(x)}_{\varepsilon })$.

**Fig. 3:**
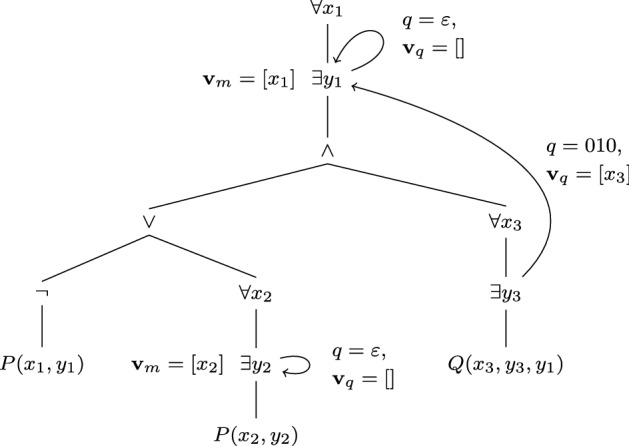
Illustration of consistent Skolemisation example

#### Example 4

Let

$$\begin{aligned} \varDelta =\forall x_1 \exists y_1. (\lnot P(x_1, y_1) \vee (\forall x_2 \exists y_2. P(x_2, y_2)) \wedge (\forall x_3 \exists y_3. Q(x_3, y_3, y_1))) \end{aligned}$$

and let $\varDelta ^n$ denote the subformula $\exists y_n. \dots$ in $\varDelta$. The formula $\varDelta$ is illustrated in Fig. 3, where an arrow from $\varDelta ^i$ to a formula *F* signifies that *F* is the $F_{\min }$ corresponding to $\varDelta ^i$, and the arrow labels *q* and $\mathbf{v}_q$ are *q* and $\mathbf{v}_q$ corresponding to $\varDelta ^i$. We then see that $F_{\min }$ for $\varDelta ^1$ and $\varDelta ^3$ is $\varDelta ^1$, and $F_{\min }$ for $\varDelta ^2$ is $\varDelta ^2$. Furthermore, let $\varDelta ^n_\lambda$ and $\varDelta ^n_\alpha$ be $F_\lambda$ and $F_\alpha$ for $\varDelta ^n$, respectively. Then $\varDelta ^1_\lambda = \lambda x_1. \exists y_1. (\lnot P(x_1, y_1) \vee (\forall x_2 \exists y_2. P(x_2, y_2)) \wedge (\forall x_3 \exists y_3. Q(x_3, y_3, y_1)))$ and $\varDelta ^2_\lambda = \lambda x_2. \exists y_2. P(x_2, y_2)$. The position *q* of $\varDelta ^3$ in $\varDelta ^1$ is 010. Therefore, our consistent Skolemisation of $\varDelta$ is $\sigma \varDelta '$, where

$$\begin{aligned} \sigma&= \left\{ y_1 \rightarrow s^{\varDelta ^1_\alpha }_\varepsilon (x_1),\; y_2 \rightarrow s^{\varDelta ^2_\alpha }_\varepsilon (x_2),\; y_3 \rightarrow s^{\varDelta ^1_\alpha }_{010}(x_1, x_3) \right\} \\ \varDelta '&= \forall x_1. (P(x_1, y_1) \rightarrow (\forall x_2. P(x_2, y_2)) \wedge (\forall x_3. Q(x_3, y_3, y_1))) \end{aligned}$$

The combination of $F_\alpha$ and *q* in the newly introduced Skolem function name allows the reconstruction of the epsilon term which epsilon-Skolemisation would have introduced for *x*. We can expand $s^{F_\alpha }_q(\mathbf{x})$ with $F_\alpha = \lambda \mathbf{v}_m. F_{\min }$ to its corresponding Skolem epsilon term by performing epsilon-Skolemisation of $F_{\min }$, obtaining the epsilon term *t* that was introduced for the variable that is existentially quantified at position *q* in $F_{\min }$, and instantiating the free variables of *t* by beta-reducing $(\lambda \mathbf{v}_m. \lambda \mathbf{v}_q. t) \mathbf{x}$, where $\mathbf{v}_q = {{\,\mathrm{\mathcal {V}ar}\,}}_{\forall }(F_{\min }, q) \cap {{\,\mathrm{\mathcal {FV}ar}\,}}(F_{\min }|_q)$.

Encoding $F_\alpha$ and *q* in the name of a first-order function symbol avoids dedicated procedures during proof search to decide the equivalence of Skolem functions and does not require adaptations of the machine learning methods to account for Skolem functions. The resulting Skolem function names are linear in the size of $\varDelta$.

### Connection Search

We explain how the prover efficiently searches for connections that correspond to extension steps. For this, let us introduce the concept of a *contrapositive*.

#### Definition 2

(*Clausal Contrapositive*) Given a clausal matrix *M* with $C \in M$ and $L \in C$, the formula $\overline{L} \rightarrow C {\setminus } \left\{ L\right\}$ is a *contrapositive* of *M*.

To find a connection with a literal *L*, it suffices to find a contrapositive of *M* with an antecedent $L'$ such that *L* and $L'$ can be unified. The consequent of the contrapositive can then be used to generate extension clauses.

#### Example 5

Consider the matrix $M'$ from Example 1 on page 3. A contrapositive of $M'$ is $Q \rightarrow (\lnot P(x) \vee P(sx))$. This contrapositive was used to find the connection $\left\{ Q, \lnot Q\right\}$ and to generate the corresponding extension clause $\left\{ \lnot P(x'), P(sx')\right\}$ in Fig. 2.

The original versions of leanCoP and nanoCoP rely on Prolog’s internal literal indexing to keep a contrapositive database. We considered storing contrapositives in first-order term indexing structures [65]. However, the overall effect on the performance of storing contrapositives in a discrimination tree [25] on the considered datasets is minor, as unification with array substitutions (see below) is relatively fast. In our implementations, we store all contrapositives in a hash table indexed by the polarity and the predicate symbol of the antecedents. To find connections with a literal *L*, we perform two steps: First, we retrieve from the hash table all contrapositives whose antecedents have the same polarity and predicate symbol as *L*, and replace their free variables with fresh ones. Second, we return those contrapositives obtained in the first step whose antecedents can be unified with *L*.

Unification is one of the most time-consuming parts of proof search. Therefore it is crucial to represent data, including substitutions, in a way that allows efficient unification. The simplest approach to represent substitutions is to use association lists from variables to terms. This is done e.g. in the HOL Light implementation of MESON. However, as variable lookup is linear in the number of bound variables, this approach does not scale well. An improvement over this is to use tree-based maps, used for example by Metis. Both solutions however incur a significant overhead in tableaux proof search, where a single large substitution is needed. In functional languages with efficient support for arrays (e.g. the ML language family, used in many proof systems), it is more efficient to store the substitution in a single global mutable array. As variables can be represented by positive integers, the *n*th array element contains the term bound to the variable *n*. By keeping a stack of variables bound in each prover state, it is also possible to backtrack efficiently: variables removed from the top of the stack are removed from the global array. This way, backtracking can be done as if the substitution was contained in a purely functional data structure, however allowing for more efficient unification.

### Proof Search

Proof search in connection tableaux calculi is analytic, i.e. the proof tree is constructed bottom-up. As the proof search is not confluent, i.e. making a wrong choice can lead to a dead-end, backtracking is necessary for completeness. The proof tree is constructed with a depth-first strategy, which results in an incomplete proof search. To remedy this, iterative deepening is used, where the maximal path length is increased in every iteration.

The connection provers leanCoP and nanoCoP use a number of optimisation techniques, such as regularity, lemmas, and restricted backtracking [58]. When backtracking is restricted, as soon as the proof search finds some proof tree to close a branch, no other potential proof trees for that branch are considered anymore. While restricted backtracking loses completeness, it significantly increases the number of problems solved for various first-order problem classes.

Prolog allows for a very elegant and succinct implementation of proof search. First attempts to directly integrate machine learning into Prolog leanCoP have suffered from low speed [83]. Later, [38, 41] showed that implementations of leanCoP in a functional programming language allow for fast machine learning. However, implementing proof search with restricted backtracking in a functional language is not straightforward.

In this section, we discuss several implementations of a clausal prover loop that can be adapted to use restricted backtracking: The simplified version of leanCoP shown in Sect. 3.4.1 is the smallest, but also the slowest implementation. For the sake of performance comparison, we take care that all subsequent implementations perform the proof search in precisely the same order as the original Prolog implementation. We then introduce purely functional implementations in Sect. 3.4.2 using lazy lists and streams. This version slightly increases code size compared to the Prolog version, but greatly improves performance, as shown in the evaluation in Sect. 3.5. We also discuss an approach based on continuations, still purely functional, but more complicated than the stream version. In exchange, this version has slightly better performance than the stream one, likely due to not having to allocate memory for (stream) constructors. The fastest, but also most complicated implementation considered in this paper uses an explicit stack and exceptions for backtracking. However, as it proves in our evaluation just as many problems as the continuation-based version, we will only briefly discuss it.

#### Prolog

A simplified version of the original leanCoP in Prolog is given in Listing 1. We explain and relate it to the clausal connection calculus introduced in Sect. 2.

The main predicate prove(*C*, *Path*, PathLim) succeeds iff there exists a closed proof tree for $\langle C, M, Path \rangle$ with a maximal path length of PathLim. For this, prove attempts to close the proof tree for the first literal Lit of *C* in lines 4–9, and if successful, it continues with the remaining clause Cla of *C* in line 10.

Let us detail the proof search for the current literal Lit: Line 4 corresponds to the *reduction* rule: The branch is closed if the negation of Lit can be unified with a literal on the *Path*. Lines 6–8 correspond to the *extension* rule: The contrapositive database as explained in Sect. 3.3 is implemented by the predicate lit(*L*, *C*), which succeeds iff the matrix contains some clause that can be unified with $\left\{ L\right\} \cup C$. This is used to obtain some contrapositive Cla1 for the negation of Lit. If the path does not exceed the length limit (line 7), new branches are opened for Cla1 in line 8.

Backtracking is handled by the Prolog semantics: For example, if choosing the first matching contrapositive for Lit leads to the proof search getting stuck, the next contrapositive will be tried by Prolog.
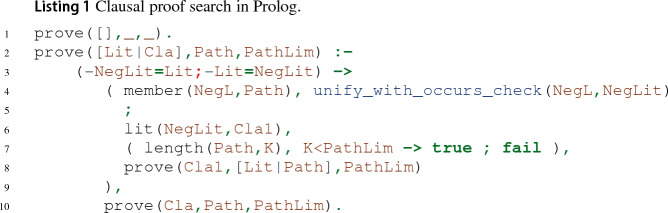


#### Lazy Lists and Streams

Proof search in a functional language can be elegantly implemented as a function from a branch to a *lazy list* of proofs, where a lazy list is an arbitrarily long list built on demand. However, as the proof search considers every list element at most once, the memoization done for lazy lists creates an unnecessary overhead. For that reason, *streams* can be used instead of lazy lists, where a stream is a special case of a lazy list that restricts list elements to be traversed at most once. As our application uses a common interface for lazy lists and streams, we solely present the lazy list version here.

Listing 2 shows a functional leanCoP implementation using lazy lists.^3^ Let us first introduce the semantics of the used constructs:

- x & f denotes f x.
- $\backslash$ x -> y stands for a lambda term $\lambda x. y$.
- unify sub lit1 lit2 unifies two literals lit1 and lit2 under a substitution sub, returning a new substitution if successful.
- unifyDB sub lit finds all contrapositives in the database which could match the literal lit under the substitution sub. It returns a list of substitution-contrapositive pairs. It corresponds to the lit predicate in the Prolog version.
- mapMaybe f l returns the results of f for the elements of l on which f succeeded.
- concatMap f l maps f over all elements of l and concatenates the resulting list of lists to form a flat list.
- x ++ y is the concatenation of two lists x and y.

The main function prove $C\ \hbox {Path}$ lim $\sigma$ returns a list of substitutions $[\sigma _1, \dots , \sigma _n]$, where every substitution $\sigma _i$ corresponds to a closed proof tree for $\langle C, M, \hbox {Path} \rangle$ with a maximal path length smaller than lim, where the global initial substitution is $\sigma$ and the final substitution is $\sigma _i$.^4^ Similarly to the Prolog version, prove attempts to close the proof tree for the first literal lit of *C* in lines 4–8, and the resulting substitutions are used to close the proof trees for the remaining clause cla of *C* in line 9. Line 4 corresponds to the reduction rule, and lines 5–8 correspond to the extension rule.^5^ As we use lazy lists / streams, a substitution $\sigma _ i$ is only calculated if proof search failed for all $\sigma _j$ with $j < i$.
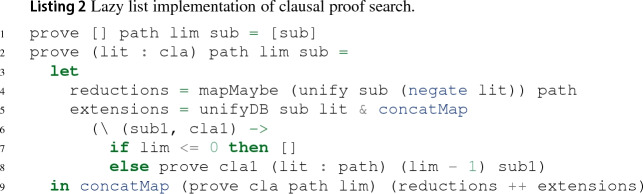


#### Continuations

Continuation passing style (CPS) allows the implementation of algorithms with complicated control flow in functional languages [64]. Listing 3 shows a leanCoP implementation using CPS. The main function prove $C\ { Path}$ lim $\sigma$ alt rem searches for a closed proof tree for $\langle C, M, Path \rangle$ with a maximal path length smaller than lim under the substitution $\sigma$. If prove finds such a proof tree, it calls the rem continuation to treat remaining proof obligations (line 1). Otherwise, prove calls the alt continuation to backtrack to an alternative (line 16). The reduce function in lines 3–7 corresponds to the reduction rule, and the extend function in lines 10–15 corresponds to the extension rule. If no more reductions can be performed, extensions are tried (line 8), and if no more extensions can be performed, we backtrack (line 16). Both reduce and extend define a continuation alt1 (line 4 and 11) to provide a way to backtrack to the current state and pass it to prove (line 7 and 15). The extend function additionally defines a continuation rem1 (line 14), which serves to continue proof search for the clause cla once a proof for the contrapositive clause cla1 was found (line 15).
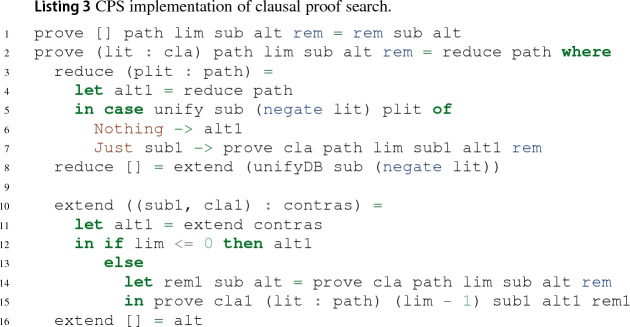


#### Stacks

The last considered implementation uses explicit stacks. There, the main prove function has the same arguments as the prove function of the stream-based implementation, plus a stack. This stack contains tuples with information about clauses that still have to be processed, together with the depth at which the clauses have been put onto the stack. Once the current clause has been completely refuted, the next tuple is popped from the stack and the clause in the tuple is processed.

### Evaluation

We evaluate the functional connection provers on several first-order problem datasets, with statistics given in Table 1:

- TPTP [76] is a large benchmark for automated theorem provers. It is used in CASC [79]. The contained problems are based on different logics and come from various domains. In our evaluation we use the nonclausal first-order problems of TPTP 6.3.0.
- MPTP2078 [2] contains 2078 problems exported from the Mizar Mathematical Library. This dataset is particularly suited for symbolic machine learning since symbols are shared between problems. It comes in the two flavours “bushy” and “chainy”: In the “chainy” dataset, every problem contains all facts stated before the problem, whereas in the “bushy” dataset, every problem contains only the Mizar premises required to prove the problem.
- Miz40 contains the problems from the Mizar library for which at least one ATP proof has been found using one of the 14 combinations of provers and premise selection methods considered in [40]. The problems are translated to untyped first-order logic using the MPTP infrastructure [81]. Symbol names are also used consistently in this dataset, and the problems are minimised using ATP-based minimisation, i.e., re-running the ATP only with the set of proof-needed axioms until this set no longer becomes smaller. This typically leads to even better axiom pruning and ATP-easier problems than in the Mizar-based pruning used for the “bushy” version above.
- HOL Light: We translate theorems proven in HOL Light to first-order logic, following a similar procedure as [37]. We export top-level theorems (“top”) as well as theorems proven by the MESON tactic (“msn”).^6^ We consider the theorems proven in the core of HOL Light (“HL”) as well as those proven by the Flyspeck project (“FS”), which finished in 2014 a formal proof of the Kepler conjecture [27].

**Table 1:** Evaluation datasets and the number of contained first-order problems

Dataset	TPTP	MPTP	Miz40	HL-top	HL-msn	FS-top	FS-msn
Problems	7492	2078	32,524	2498	1108	27,111	39,979

We use a 48-core server with AMD Opteron 6174 2.2 GHz CPUs, 320 GB RAM, and 0.5 MB L2 cache per CPU. Each problem is always assigned one CPU. We run all provers with a timeout of 10 s per problem.

We evaluate several prover configurations in Table 2. As state of the art, we use the ATPs Vampire 4.0 [45] and E 2.0 [72], which performed best in the first-order category of CASC-J8 [78]. Vampire and E are written in C++ and C, respectively, implement the superposition calculus, and perform premise selection with SInE [31]. Furthermore, Vampire integrates several SAT solvers [13], and E automatically determines proof search settings for a given problem. We ran E with --auto-schedule and Vampire with --mode casc. In addition, we evaluated the ATP Metis [33]: It implements the ordered paramodulation calculus (having inference rules for equality just like the superposition calculus), but is considerably smaller than Vampire and E and is implemented in a functional language, making it more comparable to our work.

We implemented functional-style versions of leanCoP 2.1 and nanoCoP 1.0 in the functional programming language OCaml.^7^ Our implementations use the techniques introduced such as hash-based indexing and array-based substitutions (Sect. 3.3), efficient control flow (Sect. 3.4), and consistent Skolemisation (Sect. 3.2), as well as all optimisation techniques of the Prolog implementations, such as regularity, lemmas, and restricted backtracking. We refer to our functional OCaml implementations as fleanCoP and fnanoCoP, whereas we refer to the original Prolog versions as pleanCoP and pnanoCoP. The Prolog versions were run with ECLiPSe 5.10. A prover configuration containing “$+$x” or “−x” means that feature x was enabled or disabled, respectively. “cut” denotes restricted backtracking and “conj” stands for conjecture-directed search. leanCoP was evaluated without definitional clausification, see Sect. 3.1. The OCaml implementations use streams to control backtracking (see Sect. 3.4.2) and arrays as substitutions. As strategy scheduling is not a focus of this work, we evaluate our provers with disabled strategy scheduling.

**Table 2:** Comparison of provers without machine learning

Prover	TPTP	Bushy	Chainy	Miz40	FS-top	FS-msn
Vampire	4404	1253	656	30,341	6358	39,760
E	3664	1167	287	26,003	7382	39,740
Metis	1376	500	75	18,519	3537	38,625
fleanCoP$+$cut$+$conj	1859	670	289	12,204	3980	35,738
fleanCoP$+$cut−conj	1782	598	244	11,796	3520	30,668
fleanCoP−cut$+$conj	1617	499	192	7826	3849	35,204
fleanCoP−cut−conj	1534	514	164	11,115	3492	36,334
pleanCoP$+$cut$+$conj	1673	606	182	11,243	3664	35,234
pleanCoP$+$cut−conj	1621	548	153	11,227	3305	30,416
pleanCoP−cut$+$conj	1428	453	143	7287	3671	34,437
pleanCoP−cut−conj	1374	460	123	10,442	3415	35,499
fnanoCoP$+$cut	1724	511	192	12,332	3178	30,327
fnanoCoP−cut	1567	542	151	13,316	1993	37,938
pnanoCoP$+$cut	1585	480	112	11,921	2970	30,272
pnanoCoP−cut	1485	510	126	12,943	1986	38,015

The results are shown in Table 2: The OCaml versions outperform the Prolog versions in almost all cases (the exception being fnanoCoP−cut on the FS-msn dataset). The most impressive result is achieved by fleanCoP$+$cut$+$conj on the chainy dataset: The OCaml version proves 58.8% more problems than its Prolog counterpart, thus even passing E. Furthermore, on four out of six datasets, our strongest configuration proves more problems than Metis.

nanoCoP solves more problems than leanCoP on the datasets Miz40 and FS-msn, in both cases without cut. However, for both datasets, nanoCoP proves fewer problems than any of the reference provers Vampire, E, and Metis. In conclusion, in scenarios where both Metis and leanCoP are available, the current version of nanoCoP cannot play its theoretical strength^8^ in any of the datasets evaluated.

We evaluate different proof search implementation styles in Tables 3 and 4. Here, inferences denote the number of successful unifications performed by some prover on all problems within 10 s timeout. This metric is not available for the Prolog versions, as these do not print the number of inferences performed when prematurely terminated.

To measure the impact of the substitution structure, we evaluated the best-performing implementation, i.e. the stack-based one, using a list-based substitution instead of an array-based substitution, see Table 3. This decreased the number of inferences by 50%, showing that the performance of the substitution structure is crucial for fast proof search.

**Table 3:** Impact of implementation on the efficiency of clausal proof search on the bushy MPTP2078 dataset with 10 s timeout, restricted backtracking ($+$cut), no definitional CNF, and conjecture-directed search ($+$conj)

Implementation	Solved	Inferences
Prolog	606	–
Lazy list	639	878199349
Stack (list substitution)	648	1253862954
Stream	670	1702827032
Continuation	681	2200272406
Stack	681	2490100879

**Table 4:** Impact of implementation on efficiency of nonclausal proof search on the bushy MPTP2078 dataset with 10 s timeout and restricted backtracking ($+$cut)

Implementation	Solved	Inferences
Prolog	480	–
Lazy list	504	374849495
Streams	511	495368962

## Naive Bayesian Internal Guidance

*Internal guidance* methods learn making decisions arising during proof search. Such methods do not influence decisions before proof search, such as which preprocessing options or which global strategies are used. The guided decisions have a large impact on the time required to find proofs, and in case of incomplete search strategies they determine whether a proof will be found at all. Ranking heuristics that learn from previous proofs are an example of internal guidance. In this section, we propose an internal guidance method using Naive Bayesian probability to guide connection proof search based on its intermediate proof state and previous proofs.

The assumption underlying our approach is the following: An action that was useful in a past state is likely to be useful in similar future states. What do actions, usefulness, and states signify in our setting of guiding connection proof search? We consider as action the application of the extension rule with a given contrapositive (see Sect. 3.3), because the order in which extension steps are tried has a significant effect on the performance of proof search. Furthermore, we consider an action to have been useful if the extension step ends up in the final proof. Finally, the state in which an action is performed is the proof branch in which the extension step is applied.

This assumption implies that we can estimate the usefulness of an action in a present state from the usefulness of the action in similar past states. More specifically, to estimate the usefulness of a contrapositive in the current proof branch, we can consider the usefulness of the contrapositive in similar proof branches of previous proofs. When we have a choice between different contrapositives, we can process them in order of decreasing estimated usefulness, in order to find proofs faster.

To measure the similarity between proof branches, we characterise them by *features* [42], which we explain in Sect. 4.1. In Sect. 4.2, we then calculate the utility of a contrapositive in the current branch, given knowledge about its utility in previous proofs. In Sect. 4.3, we motivate the integration of machine learning methods in the prover and introduce the prover FEMaLeCoP, which we evaluate in Sect. 4.4.

### Tableau Branch Characterisation

The words of the connection tableaux calculus $\langle C, M, { Path} \rangle$ correspond to a set of tableau branches sharing the active ${ Path}$. Therefore, to characterise a branch, we use as its *features* the set of symbols occurring in the active path. This does not include symbols in the substitution.^9^ We weigh the symbols by the number of times they appeared in all problems, giving higher weight to rarer symbols via *inverse document frequency* [36], as well as by the distance between the current depth and the depth the symbols were put onto the path, giving higher weight to symbols more recently processed.

### Naive Bayes

Given a set of contrapositives that are applicable in a tableau branch, we wish to obtain an ordering of the contrapositives such that trying the contrapositives in the given order minimises the time spent to find a proof. In this subsection, we show how to order the set of applicable contrapositives by a formula ${{\,\mathrm{\textsc {nb}}\,}}$ that is based on Naive Bayesian probability, as used for premise selection [40].

Adopting machine learning jargon, we will refer to contrapositives as *labels*, and we say that a label *l* co-occurred with a set of features $\mathbf{f}$ if the contrapositive *l* was used in a proof branch characterised by features $\mathbf{f}$ and *l* contributed to the final proof, and we say that *l* occurred if it co-occurred with some set of features. For this, we introduce a function *F*(*l*), which returns the multiset of sets of features that co-occurred with *l*, i.e. $F(l) = \{\mathbf{f} \mid (l, \mathbf{f}) \in S\}$. The total number of times that *l* occurred is |*F*(*l*)|.

#### Example 6

$F(l_1) = \left\{ \left\{ f_1, f_2\right\} , \left\{ f_2, f_3\right\} \right\}$ means that the label $l_1$ was used twice previously; once in a state characterised by the features $f_1$ and $f_2$, and once when features $f_2$ and $f_3$ were present.

Let $P(l_i, \mathbf{f})$ denote the probability that a label $l_i$ from a set $\mathbf{l}$ of potential labels is useful in a state characterised by features $\mathbf{f}$. Using Bayes’ theorem together with the (naive) assumption that features are statistically independent, we derive

$$\begin{aligned} P(l_i \mid \mathbf{f}) = \frac{P(l_i) P(\mathbf{f} \mid l_i)}{P(\mathbf{f})} =\frac{P(l_i)}{P(\mathbf{f})} \prod _ {f_j \in \mathbf{f}} P(f_j \mid l_i) \end{aligned}$$

To increase numerical stability, we calculate the logarithm of the probability

$$\begin{aligned} \ln P(l_i \mid \mathbf{f}) = \ln P(l_i) - \ln P(\mathbf{f}) + \sum _ {f_j \in \mathbf{f}} \ln P(f_j \mid l_i) \end{aligned}$$

In the final formula ${{\,\mathrm{\textsc {nb}}\,}}(l_i, \mathbf{f})$ to rank labels, we modify $\ln P(l_i \mid \mathbf{f})$ as follows:

- We add a term to discriminate against features not present in $\mathbf{f}$ that occurred in previous situations with the label $l_i$.
- We weigh the probability of any feature *f* by its inverse document frequency ${{\,\mathrm{idf}\,}}(f)$ to give more weight to rare features.
- We drop the term $\ln P(\mathbf{f})$, as we compare only values for fixed features $\mathbf{f}$.
- We weigh the individual parts of the sum with constants $\sigma _1$, $\sigma _2$ and $\sigma _3$.

The resulting formula is

$$\begin{aligned} {{\,\mathrm{\textsc {nb}}\,}}(l_i, \mathbf{f})&=\sigma _1 \ln P(l_i) \\&\quad +\sigma _2 \sum _ {f_j \in \mathbf{f}} {{\,\mathrm{idf}\,}}(f_j) \ln P(f_j \mid l_i) \\&\quad +\sigma _3 \sum _ {f_j \in \bigcup F(l_i) {\setminus } \mathbf{f}} {{\,\mathrm{idf}\,}}(f_j) \ln (1 - P(f_j \mid l_i)) \end{aligned}$$

The unconditional label probability $P(l_i)$ is calculated as follows:

$$\begin{aligned} P(l_i) = \frac{\left| F(l_i) \right| }{\sum _ {l_j \in \mathbf{l}} \left| F(l_j) \right| } \end{aligned}$$

In practice, as the denominator of the fraction is the same for all $l_i$, we drop it, similarly to $P(\mathbf{f})$ above.

To obtain the conditional feature probability $P(f_j \mid l_i)$, we distinguish whether a feature $f_j$ already appeared in conjunction with a label $l_i$. If so, then its probability is the ratio of the number of times $f_j$ appeared when $l_i$ was used to the number of times that $l_i$ was used. Otherwise, the probability is estimated to be a minimal constant probability $\mu$:

$$\begin{aligned} P(f_j \mid l_i) ={\left\{ \begin{array}{ll} \sum _ {\mathbf{f}' \in F(l_i)} {\mathbf{1 }}_{\mathbf{f}'}(f_j) / \left| F(l_i) \right| &{}\quad \text {if }\quad \exists \mathbf{f}' \in F(l_i). f_j \in \mathbf{f}' \\ \mu &{}\quad \text {otherwise} \end{array}\right. } \end{aligned}$$

Here, ${\mathbf{1 }}_A(x)$ denotes the indicator function that returns 1 if $x \in A$ and 0 otherwise.

### Implementations

The *Machine Learning Connection Prover* (MaLeCoP) was the first leanCoP-based system to explore the feasibility of machine-learnt internal guidance [83]. MaLeCoP relies on an external machine learning framework (using by default the SNoW system [19]), providing machine learning algorithms such as Naive Bayes and shallow neural networks based on perceptrons or winnow cells. During proof search, MaLeCoP sends features of its current branch to the framework, which orders the proof steps applicable in the current branch by their expected utility. The usage of a general framework eases experiments with different methods, but the prediction speed of MaLeCoP’s underlying advisor system together with the communication overhead is several orders of magnitude lower than the raw inference speed of leanCoP. This was to some extent countered by fast query caching mechanisms and a number of strategies trading the machine-learnt advice for raw speed, yet the real-time performance of the system remains relatively low.

This motivated the creation of the *Fairly Efficient Machine Learning Connection Prover* (FEMaLeCoP), which improved speed by integrating a fast and optimised Naive Bayes classifier as shown in Sect. 4.2 into the prover [38]. Naive Bayes was chosen because learning data can be easily filtered for the current problem, making the calculation of Naive Bayesian probabilities for a given branch efficient for each applicable contrapositive. FEMaLeCoP efficiently calculates the Bayesian probabilities of a given set of contrapositives by saving statistics directly in the contrapositive database, see Sect. 3.3. Performance is further improved by updating branch features from the previous branch, instead of fully recalculating them in every new branch.

### Evaluation

The evaluation of Naive Bayes guidance (as well as the comparative evaluation of other methods in the next section) will involve collecting training data by running leanCoP on a training dataset followed by running guided FEMaLeCoP both on training data and on a testing set. Additionally, to maximize the amount of available training data, we will split the dataset in such a way that problems that unmodified leanCoP can solve will be in the training set and unsolved problems will be in the testing set. We run both leanCoP and FEMaLeCoP on the bushy MPTP2078 dataset with a timeout of 60 s, use nondefinitional clausification, conjecture-directed search and restricted backtracking. Both leanCoP and FEMaLeCoP considered in this evaluation are implemented in OCaml using continuation passing style and array-based substitutions, see Sects. 3.4.3 and 3.3.

The original leanCoP orders the input formula so that more promising (e.g. smaller) clauses are tried earlier, see Sect. 3.1. To evaluate the ability of FEMaLeCoP to learn useful clause orders itself, we evaluate versions of leanCoP and FEMaLeCoP that either order the clauses like the original leanCoP or reverse the original order of clauses in the matrix. The latter reduces the number of proven problems compared to the default clause order.

Our evaluation proceeds as follows: We first run leanCoP on all problems. This divides our problems into a training set, namely the problems that leanCoP solves, and a testing set, namely the problems that leanCoP does not solve. From the proofs for the problems in the training set, we extract the information which contrapositive contributed in which tableau branch. We combine this information for all proofs in a format that allows efficient retrieval of learning data for given contrapositives. With the training data generated from the leanCoP proofs, we run FEMaLeCoP on both training and testing set.

**Table 5:** FEMaLeCoP results, reversed clause order

Prover	Training	Testing	$\sum$	$\bigcup$
leanCoP−def	574	0	574	664 ($+\mathbf{15}.7 \%$)
FEMaLeCoP−def	550 ($-$ 4.2%)	90	640 ($+\mathbf{11}.5 \%$)	
leanCoP$+$def	568	0	568	623 ($+9.7\%$)
FEMaLeCoP$+$def	540 ($-$ 4.9%)	55	595 ($+4.8\%$)	

Provers run with 60 s timeout and restricted backtracking ($+$cut)

**Table 6:** FEMaLeCoP results, default clause order

Prover	Training	Testing	$\sum$	$\bigcup$
leanCoP−def	643	0	643	701 ($+\mathbf{9}.0 \%$)
FEMaLeCoP−def	607 ($-$5.6%)	58	665 ($+3.4\%$)	
leanCoP$+$def	577	0	577	627 ($+8.7\%$)
FEMaLeCoP$+$def	542 ($-$6.1%)	50	592 ($+2.6\%$)	

Provers run with 60 s timeout and restricted backtracking ($+$cut)

The results of the evaluation with reversed and default clause order are shown in Tables 5 and 6, respectively. The $\sum$ column shows for every prover how many problems it solved in total (i.e. the sum of training and testing problems solved by the prover). The $\bigcup$ column shows how many problems were solved by either the underlying prover gathering data or the machine learning guided prover (i.e. the sum of training problems solved by the unguided prover and testing problems solved by the guided prover).

We detail the results of leanCoP and FEMaLeCoP without definitional clausification and with the reversed clause order. In this setting, leanCoP proves 574 problems. Running FEMaleCoP on this training set proves 550 problems, which is a loss of 4.2% compared to leanCoP. However, on the testing set, FEMaLeCoP proves 90 problems that were unsolved by leanCoP. Combining the problems from the training and testing set, FEMaLeCoP proves 640 problems, which is 11.5% more problems than solved by leanCoP, despite the fact that the inference rate of FEMaLeCoP is about 40% below leanCoP. The union of leanCoP and FEMaLeCoP proves 664 problems, adding 90 problems (15.7%) to the problems solved by leanCoP.

In comparison, when using versions of leanCoP and FEMaLeCoP that use the default clause order or definitional clausification ($+$def), the gain of proven problems for FEMaLeCoP is lower.

In the next section, we will show another machine learning method and compare its performance with the performance of Naive Bayesian guidance.

## Monte Carlo Proof Search

Current automated theorem provers are still weak at finding more complicated proofs, especially over large formal developments [82]. The search typically blows up after several seconds, making the chance of finding proofs in longer times exponentially decreasing [1]. This behaviour is reminiscent of poorly guided search in games such as chess and Go. The number of all possible variants there typically also grows exponentially, and intelligent guiding methods are needed to focus on exploring the most promising moves and positions.

The guiding method that has recently very significantly improved automatic game play is Monte Carlo Tree Search (MCTS), i.e. expanding the search tree based on its (variously guided) random sampling [17]. MCTS has been found to produce state-of-the-art players for several games, most notably for the two-player game Go [73], but also for single-player games such as SameGame [70] and the NP-hard Morpion Solitaire [69].

Theorem proving can be seen as a game. For instance, it has been modelled as a two-player game in the framework of game-theoretical semantics [30], but it can also be seen as a combinatorial single-player game. As shown for example in the AlphaGo system [73], machine learning can be used to train good position evaluation heuristics even in very complicated domains that were previously thought to be solely in the realm of “human intuition”. While “finishing the randomly sampled game”—as used in the most straightforward MCTS for games—is not always possible in ATP (it would mean finishing the proof), there is a chance of learning good *proof state evaluation heuristics* that will guide MCTS for ATPs in a similar way as e.g. in AlphaGo. One-step lookahead can help Vampire proof search [32], suggesting that MCTS, whose simulation phase can be seen as multi-step lookahead, can effectively guide proof search. It therefore seems reasonable to apply MCTS to the game of theorem proving.

**Fig. 4:**
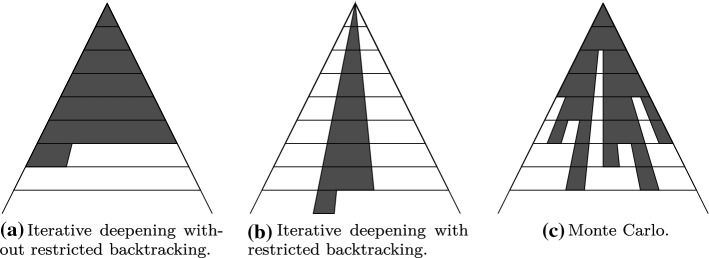
The two main leanCoP strategies compared with Monte Carlo proof search

In this section, we study MCTS methods that can guide the search in automated theorem provers. We focus on connection tableaux calculi and the leanCoP prover as introduced in Sect. 3.3. For an intuition of the relationship between different proof search strategies, see Fig. 4: Iterative deepening considers all potential proof trees of a certain depth before considering trees of higher depth. Restricted backtracking uniformly discards a set of potential proof trees. MCTS allows for a more fine-grained proof search, searching different regions of the space more profoundly than others, based on heuristics. To our knowledge, our approach is the first to apply MCTS to theorem proving.

We introduce MCTS in Sect. 5.1 and then propose a set of heuristics adapted to proof search to expand of a proof search tree using MCTS. We show an implementation in Sect. 5.6 and evaluate it in Sect. 5.8.

### Monte Carlo Tree Search

Monte Carlo Tree Search (MCTS) is a method to search potentially infinite trees by sampling random tree paths (called *simulations*) [17]. The outcome of simulations is used to estimate the quality of tree nodes, and MCTS steers search towards nodes with higher quality estimates.

#### Definition 3

(*Tree*) A *tree* is a tuple $(N, n_0, \rightarrow )$, where *N* is a set of tree nodes, $n_0 \in N$ is the root node, and $\rightarrow \; \in N \times N$ is a cycle-free relation, i.e. there is no $n \in N$ such that $n \rightarrow ^+ n$. We write that $n'$ is a child of *n* iff $n \rightarrow n'$, and we write that $n'$ is a descendant of *n* iff $n \rightarrow ^+ n'$. Every $n \in N$ is the child of at most one node in *N*.

We consider connection proof search as traversal of a tree that we define as follows.

#### Definition 4

(*Connection Proof Search Tree*) A *connection proof search tree* for a word $\langle C, M, Path \rangle$ is a tree $(N, n_0, \rightarrow )$, where *N* is the set of derivations, $n_0$ is a derivation consisting of the word $\langle C, M, Path \rangle$, and $n \rightarrow n'$ iff $n'$ can be obtained from *n* by a single application of a calculus rule. If $n \rightarrow n'$ by an application of the extension rule using the contrapositive *c*, then we write $n \xrightarrow {{{\,\mathrm{\text {ext}}\,}}(c)} n'$.

The search for a proof of the word $\langle C, M, Path \rangle$ then succeeds if we find a node $n \in N$ with $n_0 \rightarrow ^* n$ such that *n* is a closed derivation, where $(N, n_0, \rightarrow )$ is the connection proof search tree for $\langle C, M, Path \rangle$.

Let $\rho \in N \rightarrow {\mathbb {R}}$ be a *reward function* that estimates the distance of an unclosed derivation in the proof search tree from a closed derivation. Then we can use Monte Carlo Tree Search to traverse the proof search tree, giving preference to regions that yield higher rewards. For this, we first define Monte Carlo trees:

#### Definition 5

(*Monte Carlo Tree*) A *Monte Carlo tree* *T* for a tree $(N, n_0, \rightarrow )$ is a tuple $(N_T, \rightarrow _T, \rho _T)$, where $N_T \subseteq N$, $\rightarrow _T \, \subseteq \, \rightarrow ^+$, and $\rho _T \in N \rightarrow {\mathbb {R}}$ is a mapping. We write that $n'$ is a *T*-child of *n* iff $n \rightarrow _T n'$. The *initial Monte Carlo tree* $T_0$ is $(N_{T_0}, \rightarrow _{T_0}, \rho _{T_0})$ with $N_{T_0} = \{n_0\}$, $\rightarrow _{T_0} = \emptyset$ and $\rho _{T_0}(n) = 0$ for all *n*.

A single iteration of Monte Carlo Tree Search takes a Monte Carlo tree *T* and returns a new tree $T'$ as follows^10^:

1. Selection: A node $n \in N_T$ with $n_0 \rightarrow ^* _T n$ is chosen with a *child selection policy*, see Sect. 5.2.
2. Simulation: A child $n_1$ of *n* is randomly chosen with child probability $P(n_1 \mid n)$ to be the *simulation root*, see Sect. 5.3. Every tree node is chosen at most once to be a simulation root, to guarantee the exploration of the tree. From $n_1$, a sequence of random transitions $n_1 \rightarrow \dots \rightarrow n_s$ is performed, where for every $i < s$, $n_{i+1}$ is randomly selected with child probability $P(n_{i+1} \mid n_i)$.
3. Expansion: A node $n_e$ from $n_1 \rightarrow \dots \rightarrow n_s$ is selected with the *expansion policy*, see Sect. 5.5. The node $n_e$ is added as a child to *n* with reward $\rho (n_s)$ (see Sect. 5.4) to yield the new tree $T'$:
  $$\begin{aligned} N_{T'} = N_T \cup \{n_e\} \qquad \rightarrow _{T'} \; = \; \rightarrow _T \cup \; \{(n, n_e)\} \qquad \rho _{T'} = \rho _T \{n_e \mapsto \rho (n_s)\} \end{aligned}$$

In the next sections, we propose heuristics for the child selection policy, child probability, reward, and expansion policy.

### Child Selection Policy

UCT (Upper Confidence Bounds for Trees) is a frequently used child selection policy for Monte Carlo Tree Search [44]. It uses ${{\,\mathrm{\text {visits}}\,}}_T(n)$, which is the number of *T*-descendants of *n*, and $\overline{\rho } _T(n)$, which is the average *T*-descendant reward of *n*.

$$\begin{aligned} {{\,\mathrm{\text {visits}}\,}}_T(n) = |\{n' \mid n \rightarrow ^+_T n' \}| \qquad \qquad \overline{\rho }_T(n) = \frac{\sum \{ \rho _T(n') \mid n \rightarrow ^*_T n' \}}{{{\,\mathrm{\text {visits}}\,}}_T(n)} \end{aligned}$$

Given a node *n*, UCT ranks every *T*-child $n'$ of *n* with

$$\begin{aligned} {{\,\mathrm{\text {uct}}\,}}(n, n') = \overline{\rho } _T(n') + C_p \sqrt{\frac{\ln {{\,\mathrm{\text {visits}}\,}}_T(n)}{{{\,\mathrm{\text {visits}}\,}}_T(n')}} \end{aligned}$$

Here, $C_p$ is called the *exploration constant*, where small values of $C_p$ prefer nodes with higher average descendant reward and large values of $C_p$ prefer nodes with fewer visits. In the UCT formula, division by zero is expected to yield $\infty$, so if a node *n* has unvisited children, one of them will be selected by UCT.

The UCT child selection policy $cs_T(n)$ recursively traverses the Monte Carlo tree *T* starting from the root $n_0$. $cs_T(n)$ chooses the *T*-child of *n* with maximal UCT value and recurses unless *n* has no *T*-child, in which case *n* is returned:

$$\begin{aligned} cs_T(n) = {\left\{ \begin{array}{ll} \displaystyle cs_T\left( \mathop {\mathrm{arg}\,\mathrm{max}}\limits _{n' \in \{n' \mid n \rightarrow _T n'\}} {{\,\mathrm{\text {uct}}\,}}(n, n') \right) &{}\quad \text {if }\quad \exists n'. \; n \rightarrow _T n' \\ n &{}\quad \text {otherwise} \end{array}\right. } \end{aligned}$$

### Child Probability

The child probability $P(n' \mid n)$ determines the likelihood of choosing a child node $n'$ of *n* in a simulation. We show three different methods to calculate the child probability.

- The *baseline probability* assigns equal probability to all children, i.e. $P\left( n' \mid n\right) \propto 1$.
- The *open branches probability* steers proof search towards derivations with fewer open branches, by assigning to $n'$ a probability inversely proportional to the number of open branches in $n'$. Therefore, $P(n' \mid n) \propto 1 / \left( 1 + |b_o(n')|\right)$, where $b_o(n)$ returns the open branches in *n*.
- The *Naive Bayes probability* attributes to $n'$ a probability depending on the calculus rule applied to obtain $n'$ from *n*. In case the extension rule was not used, the node obtains a constant probability. If the extension rule was used, the formula ${{\,\mathrm{\textsc {nb}}\,}}$ introduced in Sect. 4.2 is used, requiring contrapositive statistics from previous proofs. However, as ${{\,\mathrm{\textsc {nb}}\,}}$ does not return probabilities, we use it to rank contrapositives by the number of contrapositives with larger values of ${{\,\mathrm{\textsc {nb}}\,}}$:
  $$\begin{aligned} {{\,\mathrm{\text {rank}}\,}}_{{{\,\mathrm{\textsc {nb}}\,}}}(n, c) = \left| \left\{ c' \mid n \xrightarrow {{{\,\mathrm{\text {ext}}\,}}(c')} n', {{\,\mathrm{\textsc {nb}}\,}}(c', \mathbf{f}(n)) \ge {{\,\mathrm{\textsc {nb}}\,}}(c, \mathbf{f}(n)) \right\} \right| , \end{aligned}$$
  where $\mathbf{f}(n)$ denotes the features of the derivation *n*. Then, we assign to nodes as probability the inverse of the Naive Bayes rank:
  $$\begin{aligned} P(n' \mid n) \propto {\left\{ \begin{array}{ll} 1 / {{\,\mathrm{\text {rank}}\,}}_{{{\,\mathrm{\textsc {nb}}\,}}}(n, c) &{} \quad \text {if }\quad n \xrightarrow {{{\,\mathrm{\text {ext}}\,}}(c)} n' \\ 1 &{} \quad \text {otherwise} \end{array}\right. } \end{aligned}$$

### Reward

The reward heuristic estimates the likelihood of a given derivation to be closable. In contrast, most prover heuristics (such as child probability) only compare the quality of children of the same node. We use our reward heuristics to evaluate the last node *n* of a simulation.

Several heuristics in this section require a normalisation function, for which we use a strictly increasing function ${{\,\mathrm{\text {norm}}\,}}\in [0, \infty ) \rightarrow [0, 1)$ that fulfils $\lim _{x \rightarrow \infty } {{\,\mathrm{\text {norm}}\,}}(x) = 1$ and ${{\,\mathrm{\text {norm}}\,}}(0) = 0$. For example, ${{\,\mathrm{\text {norm}}\,}}(x) = 1 - (x + 1)^{-1}$.

- The *branch ratio reward* determines the reward to be the ratio of the number of closed branches and the total number of branches, i.e. $\rho (n) = |b_c(n)| / |b(n)|$.
- The *branch weight reward* is based on the idea that many open branches with large literals are indicators of a bad proof attempt. Here, the size |*l*| of a literal is measured by the number of symbol occurrences in *l*. Furthermore, the closer to the derivation root a literal appears, the more characteristic we consider it to be for the derivation. Therefore, the reward is the average of the inverse size of the branch leaves, where every leaf is weighted with the normalised depth of its branch.
  $$\begin{aligned} \rho (n) = \frac{1}{|b_o(n)|} \sum _{b \in b_o(n)} \frac{{{\,\mathrm{\text {norm}}\,}}({{\,\mathrm{\text {depth}}\,}}(b))}{|{{\,\mathrm{\text {leaf}}\,}}(b)|} \end{aligned}$$
- The *machine-learnt closability reward* assumes that the success ratio of closing a branch in previous derivations can be used to estimate the probability that a branch can be closed in the current derivation. This needs the information about attempted branches in previous derivations, and which of these attempts were successful. We say that a literal *l* stemming from a clause *c* is attempted to be closed during proof search when *l* lies on some branch. The attempt is successful iff proof search manages to close all branches going through *l*. Given such data from previous proof searches, let *p*(*l*) and *n*(*l*) denote the number of attempts to close *l* that were successful and unsuccessful, respectively. We define the *unclosability* of a literal *l* as $\frac{n(l)}{p(l) + n(l)}$. However, the less data we have about a literal, the less meaningful our statistics will be. To account for this, we introduce *weighted unclosability*: We assume that a literal that never appeared in previous proof searches is most likely closable, i.e. its weighted unclosability is 0. The more often a literal was attempted to be closed, the more its weighted unclosability should converge towards its (basic) unclosability. Therefore, we model the probability of *l* to be closable as
  $$\begin{aligned} P(l \, {{\,\mathrm{\text {closable}}\,}}) = 1 - {{\,\mathrm{\text {norm}}\,}}(p(l)+n(l)) \frac{n(l)}{p(l)+n(l)} \end{aligned}$$
  Finally, the closability of a derivation is the mean closability of all leafs of open branches of the derivation, i.e. the final reward formula is
  $$\begin{aligned} \rho (n) = \sum _{b \in b_o(n)} \frac{P({{\,\mathrm{\text {leaf}}\,}}(b)\, {{\,\mathrm{\text {closable}}\,}})}{|b_o(n)|} \end{aligned}$$

To measure the efficiency of a reward heuristic, we introduce *discrimination*: Assume that an MCTS iteration of the Monte Carlo tree *T* starts a simulation from the node $n_p$ and finds a proof. Then the discrimination of *T* is the ratio of the average reward on the Monte Carlo tree branch from the root node $n_0$ to $n_p$ and the average reward of all Monte Carlo tree nodes. Formally, let the average reward of a set of nodes *N* be

$$\begin{aligned} \overline{\rho } _T(N) = \frac{\sum \left\{ \rho _T(n) \mid n \in N\right\} }{|N|} \end{aligned}$$

Then, the discrimination of *T* is

$$\begin{aligned} \frac{\overline{\rho } _T(\{ n \mid n_0 \rightarrow _T^* n, n \rightarrow _T^* n_p \})}{\overline{\rho } _T(\{ n \mid n_0 \rightarrow _T^* n \})} \end{aligned}$$

### Expansion Policy

The expansion policy determines which node $n_e$ of a simulation $n_1 \rightarrow \dots \rightarrow n_s$ is added to the Monte Carlo tree. We implement two different expansion policies:

- The *default expansion policy* adds $n_1$, i.e. the simulation root, to the MC tree.
- The *minimal expansion policy* picks $n_e$ to be the smallest of the simulation nodes with respect to a given norm $|\cdot |$, such that for all *i*, $|n_e| \le |n_i|$. If multiple $n_e$ are admissible, the one with the smallest index *e* is picked. We consider two norms on nodes:
  1. The first norm measures the number of open branches.
  2. The second norm measures the sum of depths of open branches.

The minimal expansion policy is similar to restricted backtracking in the sense that it restricts proof search to be resumed only from certain states, thus resulting in an incomplete search.

### Implementation

We implemented Monte Carlo proof search (MCPS) on top of the functional implementation of leanCoP using lazy lists, see Sect. 3.4.2.^11^ In our implementation, leanCoP provides the search tree and MCTS chooses which regions of the tree to search. Unlike for the traditional leanCoP, the depth of the search tree is not limited. To guarantee nonetheless that simulations terminate, simulations are stopped after a fixed number of simulation steps $s_{\max }$.

While it is possible to run MCPS from the root node until a proof is found, we found it to perform better when it serves as *advisor* for leanCoP. We show this in Listing 4, assuming for a simpler presentation that the default expansion policy from Sect. 5.5 is used: In line 4, initTree $L\ { Path}\ \sigma$ creates an initial Monte Carlo tree for a connection proof search tree for the word $\langle \{L\}, M, { Path} \rangle$ under the substitution $\sigma$. Starting from this initial Monte Carlo tree *T*, mcps *T* constructs a (potentially infinite) lazy list of Monte Carlo iterations, with *T* as its head, where an iteration consists of a Monte Carlo tree and possibly a proof discovered during the simulation performed in the iteration. Of this list, we consider *T* and the following maxIterations elements: When maxIterations is set to 0, only *T* is considered and thus proof search behaves like leanCoP. When maxIterations is set to $\infty$, the whole proof search is performed in the MCPS part. As MCPS is performed lazily, MCPS may be performed for less than maxIterations iterations when it discovers some proof contributing to the final closed derivation. Here, the lazy list characterisation introduced in Sect. 3.4.2 turns out to permit a very concise implementation as well as an easy integration of techniques such as restricted backtracking. As soon as all proofs discovered during MCPS were considered (line 5), the tree *T* of the final Monte Carlo iteration last mc is obtained and the children of the root of *T* are sorted by decreasing average *T*-descendant reward $\overline{\rho } _T$ (line 6). Finally, the last applied proof step of each child is processed like in the lazy list implementation (lines 7–11).
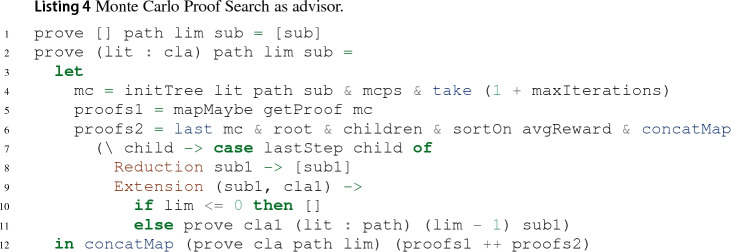


The array substitution technique from Sect. 3.3 requires that the proof search backtracks only to states whose substitution is a subset of the current state’s substitution. However, because this requirement is not fulfilled for MCPS, we use association lists for substitutions.

### Parameter Tuning

To obtain suitable parameters for our heuristics, we evaluate them on the bushy MPTP2078 problems, with definitional clausification and a timeout of 10 s for each problem. Before evaluation, we collect training data for machine learning heuristics by running leanCoP with a strategy schedule on all bushy problems with a timeout of 60 s. This solves 600 problems.

The base configuration of monteCoP uses the open branches probability (see Sect. 5.3), the branch ratio reward (see Sect. 5.4), and the minimal expansion policy 1 (see Sect. 5.5), where the maximal simulation depth $s_{\max } = 50$, the exploration constant $C_p = 1$, and the maximal number of MCTS iterations maxIterations $= \infty$. For any heuristic *h* not used in the base configuration, we replace the default heuristic with *h* and evaluate the resulting configuration. The results are shown in Table 7: The heuristics that most improve the base configuration are the machine-learnt closability reward and the minimal expansion policy 2.

**Table 7:** Comparison of Monte Carlo heuristics

Configuration	Iterations	Sim. steps	Discr.	Solved
Base	116.46	1389.82	1.37	332
Uniform probability	949.62	17,539.59	1.31	237
NB probability	528.39	8014.03	1.35	248
Random reward	104.88	1167.98	1.19	364
Branch weight reward	108.13	1268.88	1.12	334
ML closability reward	108.52	1151.61	**2.30**	**367**
Default exp. pol.	371.81	4793.58	1.38	328
Minimal exp. pol. 2	224.72	2769.12	1.40	348

Iterations, simulation steps and discrimination ratio are averages on the 196 problems solved by all configurations

**Fig. 5:**
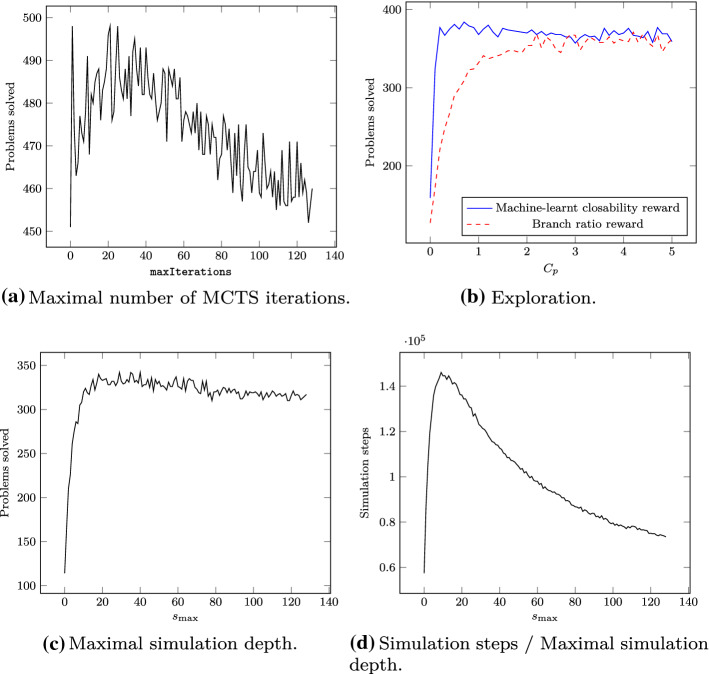
Parameter influence

We explore a range of values for several numeric parameters, for which we show results in Fig. 5: The maximal number of MCTS iterations maxIterations performs best between 20 and 40, see Fig. 5a: Below 20, MCTS cannot provide any meaningful quality estimates, and above 40, the quality estimates do not significantly improve any more, while costing computational resources. The exploration constant $C_p \approx 0.75$ gives best results, where the machine-learnt closability reward achieves a local optimum, see Fig. 5b: At such an optimum, exploration and exploitation combine each other best, therefore the existence of such an optimum is a sanity check for reward heuristics (which the branch ratio reward does not pass). The maximal simulation depth $s_{\max } \approx 20$ seems to perform best, see Fig. 5c. Above this value, the number of solved problems decreases, since the number of actually performed simulation steps decreases, as shown in Fig. 5d. This might be explained by the fact that at higher simulation depths, the computational effort to calculate the set of possible steps increases, for example because the substitution contains more and larger elements.

We adapt the base configuration to use the best heuristics from Table 7 and the best values for parameters discussed in Fig. 5, yielding $s_{\max } =20$, $C_p = 0.75$, and maxIterations $= 27$. We use this improved configuration as basis for the following evaluation.

### Evaluation

We now compare the performance of FEMaLeCoP with default clause order and the improved configuration of monteCoP obtained in Sect. 5.7. In the following, leanCoP/m and leanCoP/F refer to the leanCoP versions used to generate training data for monteCoP and FEMaLeCoP, respectively. We use the same evaluation methodology as in in Sect. 4.4: First, we run leanCoP/m and leanCoP/F on all problems for 60 s each, collecting training data. Next, to maximize the amount of available training data, we split the dataset for both leanCoP/m and leanCoP/F in such a way that problems that the respective prover can solve will be in its training set and unsolved problems will be in its testing set. Then, we run FEMaLeCoP and monteCoP on the testing sets corresponding to their respective underlying leanCoP versions, again for 60 s each.

With a timeout of 60 s, monteCoP solves 601 problems, compared to 563 solved by the best single leanCoP/m strategy, see Table 8. In comparison, FEMaLeCoP solves 592 problems, compared to 577 solved by the best single leanCoP/F strategy, see Table 6.

**Table 8:** Final evaluation results

Prover	Training	Testing	$\sum$	$\bigcup$
leanCoP/m	563	0	563	653 ($+\mathbf{16}.0 \%$)
monteCoP	511 ($-$ 9.2%)	90	601 ($+6.7\%$)	
leanCoP/F	577	0	577	627 (+8.7%)
FEMaLeCoP$+$def	542 ($-$ 6.1%)	50	592 ($+2.6\%$)	

Provers run with 60 s timeout, definitional clausification ($+$def), and restricted backtracking ($+$cut). The $\sum$ and $\bigcup$ columns are explained in Sect. 4.4

**Fig. 6:**
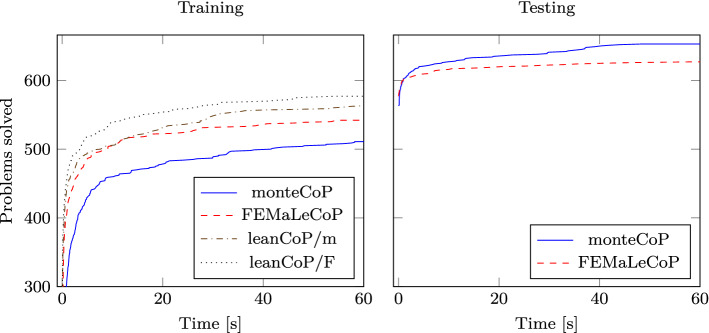
Comparison of monteCoP and FEMaLeCoP. Provers run with definitional clausification ($+$def) and restricted backtracking ($+$cut)

Figure 6 shows for any moment in time the total number of both training and testing problems solved up to that point. To this end, the testing data graph offsets the curves for monteCoP and FEMaLeCoP by the number of problems solved during training by leanCoP/m and leanCoP/F, respectively. Furthermore, we show on the training graph the number of training problems solved by monteCoP and FEMaLeCoP using training data from leanCoP/m and leanCoP/F.

On Fig. 6, we can see that despite monteCoP’s poor performance on the training set and despite the lower amount of problems solved by leanCoP/m compared to leanCoP/F, monteCoP is quickly taking the lead on the testing set compared to FEMaLeCoP. In total, the combination of leanCoP/F and FEMaLeCoP proves 627 problems, whereas leanCoP/m and monteCoP prove 653 problems. That means that in the evaluated scenario, the combination of leanCoP/m and monteCoP is more effective than leanCoP/F and FEMaLeCoP.

## Related Work

A number of related works has already been discussed in previous sections. In particular, in Sect. 2, we introduced the connection calculus [11] as a variant of tableaux [48], we discussed its implementation in the leanCoP theorem prover [62], a number of improvements introduced in the second version of leanCoP [57] including restricted backtracking [58], and the nonclausal variant of the connection calculus [59] together with its implementation [61].

The compact Prolog implementation of theorem provers following the *lean* architecture made it attractive for many experiments both with the calculus and with the implementation. The intuitionistic version of leanCoP [56] became the state-of-art prover for first-order problems in intuitionistic logic [67]. Connections have also been considered for first-order modal logic in mleanCoP [60], for higher-order logic [3] and for linear logic [23]. Various implementation modifications can be performed very elegantly, such as search strategies, scheduling, randomization of the order of proof search steps [66], and internal guidance [38, 83].

A number of early learning and data based approaches to guide automated theorem provers has been surveyed in [20]. The Prover9 hints method [85] allows the user to specify (an often large set of) clauses to treat in a special way. A similarly working *watch list* has been later integrated in E, along with other learning mechanisms [71]. Using machine learning for internal guidance is historically motivated by the success of the *external guidance* methods used mainly for premise selection outside of the core ATP systems [14, 37, 84]. Guiding the actual proof search of ATPs using machine learning has been considered in the integration of a Naive Bayesian classifier to select next proof actions in Satallax [21], as well as in Enigma [35] where the clause selection in E uses a tree-based n-gram approach to approximate similarity to the learned proofs using a support vector machine classifier. Holophrasm [87] introduces a theorem prover architecture using GRU neural networks to guide the proof search of a tableaux style proof process of MetaMath. TensorFlow neural network guidance was integrated in E [50], showing that with batching and hybrid heuristics, it can solve a number of problems other strategies cannot solve. Finally, various reasons as to why the connection calculus is well suited for machine learning techniques, especially deep learning, are considered in [12].

The main use of machine learning in automated and interactive theorem provers today is to reduce original problems before the actual proof search. Machine learning based methods [15, 47] improve on and complement the various ATP heuristics [31] and ITP heuristics [52]. The problem of selecting the most useful lemmas for the given proof, referred to as “premise selection” or “relevance filtering” [2] nowadays uses syntactic similarity approaches, simple Naive Bayes and k-NN based classifiers, regression and kernel based methods [46], as well as deep neural networks [34]. This has become especially important in the “large theory bench” division added to the CADE Automated Systems Competition in 2008 [75], with systems such as MaLARea [84] and ET [43] achieving notable results.

## Conclusion and Future work

We have presented our framework for integrating machine learning in connection tableaux. First, we presented translations to functional programming languages, exploring possibilities to increase the speed of proof search while keeping the implementation as simple as possible. We showed that the number of solved problems can be increased by up to 58.8%, on one dataset beating even E in automatic mode. Then, we discussed machine learning integration in leanCoP via context-sensitive clause ordering and Monte Carlo Tree Search, showing that both these techniques can increase the number of solved problems, despite fewer inferences being performed.

The performed machine learning experiments are promising enough to justify the enhancement of Monte Carlo Proof Search with stronger heuristics, such as neural networks. While we applied Monte Carlo Tree Search to theorem proving as a single-player game, it could also be used to treat theorem proving as a two-player game.

The combination of several tools that are small, simple and comprehensible can be more effective than a large, monolithic tool. While the resulting connection provers cannot yet outperform larger systems like Vampire [45] and E [72], we hope that the insight gained by experiments performed in connection provers might be used in their complex counterparts. Connection provers might be candidates for the core of future automated reasoning tools and artificial intelligence experiments.

## Footnotes

1 The source code of all implementations in this article is available at http://cl-informatik.uibk.ac.at/users/mfaerber/cop.html.

2 The substitution $\sigma$ maps existentially quantified variables to their respective Skolem terms. The new substitution $\sigma '$ is a strict superset of $\sigma$ because it preserves all previously established mappings to Skolem terms as well as adds a new mapping from the variable *x* to some Skolem term.

3 Several of the algorithms shown in this paper rely on lazy evaluation. Therefore, we show Haskell versions of our algorithms, which are shorter than those in our actual implementation language OCaml.

4 In this simplified implementation, the actual proof tree is not recorded, in contrast to our actual implementation. The same holds for the Prolog version.

5 The shown program could be easily improved, for example by moving the check lim $\texttt {<=}$ 0 from line 7 to line 5. However, the actual implementation performs at this place a more complex check which cannot be moved this way. Therefore we leave the check here as it is.

6 As part of exporting theorems solved by MESON, we perform some of the original MESON preprocessing, such as propositional simplification, Skolemisation, currying and so on. This preprocessing may solve the problem, in which case we do not export the problem at hand.

7 The source code is available at http://cl-informatik.uibk.ac.at/users/mfaerber/cop.html.

8 The nonclausal calculus underlying nanoCoP can linearly simulate the clausal calculus underlying leanCoP, but there exist nonclausal proofs of which no clausal equivalent of polynomial size exists [59].

9 It is possible either to consider or to disregard symbols that are introduced by previous unifications, i.e. symbols in the global substitution. In our experiments, disregarding such symbols as features turned out to be more beneficial.

10 Frequently, MCTS is described to have a *backpropagation* step that adds rewards to the ancestors of the newly added nodes. We omit this step, adapting the child selection policy instead.

11 The source code is available at http://cl-informatik.uibk.ac.at/users/mfaerber/cop.html.
